# Psychosocial Factors of Subjective Well-Being in Women with Eating Disorders

**DOI:** 10.3390/bs13070594

**Published:** 2023-07-16

**Authors:** Helena Matkovic, Lovorka Brajkovic, Vanja Kopilaš

**Affiliations:** Department of Psychology, Faculty of Croatian Studies, University of Zagreb, 10 000 Zagreb, Croatia

**Keywords:** eating disorders, subjective well-being, loneliness, resilience

## Abstract

Eating disorders are known as the most lethal mental health conditions, and lately there has been a significant increase in the prevalence of these disorders. The aim of this research was to determine the perceived quality of professional support, the relationship between subjective well-being, loneliness, resilience, and the quality of family functioning, and the possibility of predicting subjective well-being based on knowledge of psychosocial factors in people with eating disorders. Eighty-six women with a diagnosed eating disorder participated in the online survey. The Diener Subjective Well-Being Scale, the UCLA Loneliness Scale, the Brief Resilience Scale, and the Self-Report Family Inventory were used to measure the constructs. Questions were constructed to collect information about the perceived quality and availability of professional support. Results showed lower levels of life satisfaction and flourishing and more frequent negative experiences. Reports of medium levels of loneliness, lower levels of flourishing, and lower perceived quality of family functioning were also obtained. Significant predictors of subjective well-being were loneliness and resilience, while family cohesion was significant in predicting positive and negative experiences and flourishing. These findings can contribute to the recognition of aspects existent prior to the development of the disorder, based on which experts can determine what to focus on in the treatment process.

## 1. Introduction

In recent years, the number of people with eating disorders has increased significantly. Although they are still more prevalent among women, there has been an increase in eating disorders among the male population [1]. Eating disorders became more pronounced and widespread during the COVID-19 pandemic, partly due to social distancing policies, staying at home being mandatory, and limited health services [2]. Research has shown that, during isolation, physical activity decreased, and there were changes in dietary habits (type of food consumed, uncontrolled eating, frequent snacks, change in the number of main meals) that proved to be less healthy than before isolation [3]. Increased isolation, fear of infection, reduced satisfaction with relationships with family and friends, reduced perceived social support, and increased exposure to unusual life routines have been described as possible factors contributing to the worsening of specific and general psychopathological symptoms of nutrition in people with eating disorders [4].

In the latest edition of the Diagnostic and Statistical Manual of Mental Disorders (DSM-5), eight possible diagnoses of feeding and eating disorders are listed, of which the most well-known diagnoses are anorexia nervosa, bulimia nervosa, and binge eating disorder [5]. Feeding and eating disorders, despite similar psychological and behavioral patterns, differ in clinical picture, course of treatment, and final outcome [6].

### 1.1. Subjective Well-Being and Eating Disorders

Subjective well-being refers to the affective and cognitive evaluation of one’s own life, which assesses emotional reactions in different situations, as well as cognitive judgments of satisfaction and fulfilment [7]. It can be assessed using various measures such as life satisfaction, quality of life, and life fulfilment. Subjective well-being encompasses the experience of pleasant emotions, low levels of negative moods, and high life satisfaction. Experienced positive experiences, which are part of high subjective well-being, are a key concept in positive psychology because they are considered to make life worth living [8].

Individuals diagnosed with an eating disorder report lower levels of life satisfaction than those in the general population. This is significantly associated with the individual having been diagnosed with an eating disorder in the past, specific symptoms of eating disorders, and numerous difficulties in various aspects of life [9]. Exposure to content that supports behaviors characteristic of an eating disorder (e.g., various methods of purging, starvation) is associated with lower subjective well-being and can potentially encourage even those individuals who do not have a history of eating disorders to undertake such methods [10].

It has been proven that women who are satisfied with their bodies are also more satisfied with their lives and report more frequent positive experiences, unlike women diagnosed with an eating disorder, whose life satisfaction is significantly lower [9,11]. The study showed that there is no statistically significant difference among individuals with different eating disorders in the assessment of subjective well-being, which implies that none of the eating disorders (anorexia, bulimia, and unspecified eating disorder) is less susceptible to poorer quality of life than the others [12]. Acceptance and satisfaction with one’s own body image are important predictors of life satisfaction and a sense of flourishing in both women and men [11]. Research showed that women with eating disorders experience positive experiences less frequently, but there was no statistically significant difference in the experience of negative experiences between women with eating disorders and the control group [12]. On the other hand, experiencing negative experiences such as childhood abuse and exposure to traumatic events has been identified as a possible risk factor for the development and maintenance of eating disorders [13]. During the COVID-19 pandemic, there was a worrying increase in domestic violence and child abuse because individuals’ access to resources for reporting domestic violence and abuse was limited by social distancing measures [14].

### 1.2. Psychosocial Factors of Subjective Well-Being in Individuals with Eating Disorders

There are numerous psychosocial factors that affect different aspects of subjective well-being in individuals with eating disorders, such as loneliness, resilience, family relationships, quality of social support, etc. Loneliness has been shown to be a major predictor of numerous psychological problems, such as depression, psychological stress, and anxiety [15]. Researchers found a small but significant increase in loneliness during the COVID-19 pandemic among different groups of people [16]. The relationship between loneliness and eating disorders extends across the entire spectrum of severity from anorexia nervosa to binge eating and obesity [17]. Lonelier individuals are more likely to use food as a coping mechanism for unpleasant emotions, which can result in disordered eating patterns. This behavior is most common in individuals diagnosed with binge eating disorder [18]. Inadequate emotion regulation is associated with more pronounced symptoms of bulimia and binge eating disorder in women, and this relationship is mediated by feelings of loneliness, suggesting that inadequate emotion regulation may exacerbate the symptomatology of these disorders in women who are lonelier [19]. Individuals diagnosed with an eating disorder, specifically anorexia, bulimia, or binge eating disorder, show lower levels of psychological resilience [20,21]. Resilience is significantly lower in women with eating disorders than in the general population and in those who have recovered from eating disorders [22]. Resilience has been shown to be significant in predicting quality of life in individuals with eating disorders, which may have numerous clinical implications [23]. The COVID-19 pandemic required major changes and adaptations in daily life that presented major challenges for many [24]. Such stressors affect individuals with mental disorders more than healthy individuals, who report higher levels of resilience. Accordingly, individuals diagnosed with bulimia showed a significant worsening of symptoms and quality of life during this period [25]. Strong family support has been shown to be helpful in strengthening resilience. Individuals with eating disorders report that family support and understanding from others are integral parts of their path to strengthening resilience because it encourages them to seek advice, help, and moral support [26].

Previous research has provided a family systems perspective on the role of attachment in eating disorders, suggesting that hidden family conflicts in the parent–child relationship and a history of parental trauma foster the development of insecure attachment patterns and eating disorders [27]. When family relationships are stable and supportive, an individual whose mental health is compromised or who is suffering from a specific disorder may respond better to treatment. Both peers and family can influence eating patterns, body dissatisfaction, and the emergence of bulimia symptoms in adolescents [28]. In individuals diagnosed with an eating disorder, a sense of family cohesion has a significant negative association with the severity of symptoms of a particular disorder. In addition, cohesion is negatively associated with loneliness, which is positively associated with the severity of eating disorder symptoms [29]. Individuals with anorexia and bulimia assess family relationships as less cohesive and less adaptable, while individuals with unspecified eating disorders indicate separation among family members [30]. These findings suggest that, as the sense of cohesion in the family increases, feelings of loneliness and the severity of eating disorder symptoms decrease. Numerous pathological behavior patterns in individuals can be attributed to family inheritance, which also applies to diagnoses of eating disorders. Patients with eating disorders often have poor insight into their condition and have difficulty verbalizing their own feelings, which is why they often delay seeking professional help [31].

### 1.3. The Relationship between Psychosocial Factors and Subjective Well-Being in Individuals with Eating Disorders

Positive relationships with others, self-acceptance, resilience, social and family support, and a sense of belonging contribute to a greater sense of subjective well-being in individuals with eating disorders [32]. It has been proven that social support reduces loneliness, which is negatively associated with subjective well-being, meaning that individuals who are lonelier rate their subjective well-being lower [33]. Research findings confirmed that the early family environment has an impact on the levels of positive affect that children experience in adulthood. In other words, perceived family cohesion in childhood predisposes individuals to be more prone to experiencing more positive emotions and greater life satisfaction later [34].

### 1.4. Objective of the Study

The aim of this study was to determine the perceived quality of professional support, the relationship between subjective well-being (life satisfaction, positive and negative experiences, and flourishing), loneliness, resilience, and the quality of family functioning, and the possibility of predicting subjective well-being based on knowledge of psychosocial factors in individuals with eating disorders.

## 2. Materials and Methods

### 2.1. Study Design and Setting

This was a cross-sectional study conducted on people diagnosed with eating disorders. Prior to conducting the research, the permission for this research was obtained by the Department of Psychology, Faculty of Croatian Studies at the University of Zagreb (Zagreb, Croatia). The research complied with all ethical principles. Data were collected in a Google Forms questionnaire that was disseminated from November 2022 to February 2023 through convenient sampling in the two associations and centers for individuals with eating disorders in Zagreb, Croatia. Before starting the questionnaire, participants were provided with necessary information about the purpose of the study, data anonymity, and the possibility of withdrawing from the study at any time (informed consent), and the study was conducted in accordance with all ethical principles. In addition, participants had access to the researchers’ email addresses, to which they could address any doubts or additional questions related to the study.

### 2.2. Participants

In order to meet the inclusion criteria for participation in the study, participants had to be at least 18 years old and have a clinical diagnosis of any eating disorder before the start of the COVID-19 pandemic.

### 2.3. Measures

The outcome measure of interest in this study was subjective well-being, while predictors were loneliness, resilience, and quality of family functioning.

#### 2.3.1. Subjective Well-Being Scales

Data on the subjective well-being of participants were collected using a Croatian adaptation of Diener’s scales of subjective well-being [35], assessed on three subscales—the Life Satisfaction Scale, Positive and Negative Experiences Scale, and Flourishing Scale [7]. The Life Satisfaction Scale consists of 5 items such as “If I could live my life over, I would change almost nothing”, intended to measure the cognitive evaluation of satisfaction with one’s own life. Participants rate their agreement with the items on a seven-point scale, where 1 indicates general disagreement with the statement, and 7 indicates complete agreement with the statement. By summing the ratings of the responses to all five items, a total scale score is obtained, with higher scores indicating greater life satisfaction. The Positive and Negative Experiences Scale is divided into two subscales that examine positive (e.g., “Pleasant,” “Joyful”) and negative feelings (e.g., “Scared,” “Sad”), each consisting of 6 items. Participants rate their experiences in the last four weeks on a five-point scale (from 1—very rarely to 5—very often or always). A total score is formed separately for each subscale and can vary from 6 to 30 on both scales. However, these two results can also be combined by subtracting the total score on the negative experiences scale from the total score on the positive experiences scale. The resulting score can vary from −24 to 24, with lower, i.e., negative, scores indicating more frequent positive experiences. The Flourishing Scale consists of 8 items describing different segments of human functioning, such as “My relationships with others are supportive and rewarding” and “People respect me”. Participants rate their agreement with each item on a seven-point scale (from 1—completely disagree to 7—completely agree). The total score on the scale is formed by summing all ratings and can vary from 8 to 56, with higher scores indicating greater perceived success in important areas of functioning. Reliability analysis in this study showed satisfactorily high levels of internal consistency and reliability for the Life Satisfaction Scale (α = 0.89), Positive Experiences Scale (α = 0.93), Negative Experiences Scale (α = 0.83), and Flourishing Scale (α = 0.92).

#### 2.3.2. UCLA Loneliness Scale

The UCLA Loneliness Scale consists of 20 items describing individual situations related to the state of loneliness [36]. Participants rate the frequency of each statement (e.g., “How often do you feel that you are no longer close to anyone?”) on a four-point scale (from 1—never to 4—always). Reliability analysis in this study had a high reliability coefficient (α = 0.91).

#### 2.3.3. The Brief Resilience Scale

The Brief Resilience Scale includes 6 items, of which three are positive (e.g., “It doesn’t take me long to recover from a stressful event”) and three are negative (e.g., “It’s hard for me to recover after something bad happens”) [37]. Participants rate their resilience on a five-point scale (from 1—completely disagree to 5—completely agree). Testing for internal consistency and reliability in this study yielded an alpha coefficient of 0.89. The total result on the scale is formed as the average result on all items, with the previous reverse scoring of the three items in the negative direction, where a higher result reflects a higher level of resilience.

#### 2.3.4. Self-Report Family Inventory II

The Self-Report Family Inventory consists of 36 items, and participants rate their degree of agreement on a seven-point scale (from 1—completely disagree to 7—completely agree), with higher scores reflecting a higher degree of cohesion, harmony, and tolerance, and lower scores implying a higher degree of family conflict [38]. Exploratory factor analysis revealed the existence of four factors: cohesion (10 items, e.g., “Members of my family like to participate in most activities together”), harmony (7 items, e.g., “I feel loved in my home”), tolerance (6 items, e.g., “In my family, we accept each other’s friends”), and conflict (8 items, e.g., “In my family, we are careful not to hurt each other”). Reliability coefficients in this study were: cohesion (α = 0.90), harmony (α = 0.87), tolerance (α = 0.92), and conflict (α = 0.71).

#### 2.3.5. Information on Perceived Quality of Support

For the purposes of this study, five questions were constructed to describe the perceived quality of professional support in participants with eating disorders. To the question “Are there Centers/Associations for eating disorders near you?” participants responded with “Yes”, “No”, or “I’m not sure”, and, with regard to the questions “Have you sought help/support from certain Centers/Associations?”, “How many times since the last two years have you sought help/support from certain Centers/Associations?”, “Do you think that the help of professionals has benefited you?”, and “What has helped you the most from the help/support provided by professionals, and what would you change?”, information was collected on how many of them sought professional help and whether they believed that the help provided was beneficial and in what way.

### 2.4. Bias

We made sure that all participants were provided with identical instructions and did not have direct communication with the researchers. As a result, the inadvertent transmission of researchers’ expectations regarding the outcomes was prevented, thereby reducing the probability of socially desirable responses.

### 2.5. Sample Size

G*Power software (version 3.1) was used, and the appropriate sample size for a moderately sized effect (effect size f = 0.2, α = 0.05, 1 − β = 0.8) was at least 75 participants.

### 2.6. Data Analysis

The data were analyzed using the IBM SPSS Statistics program (version 26). For testing the normality of distributions, the Kolmogorov–Smirnov test was used (Table 1), and results indicated the normality of distributions for all variables except for the variables of life satisfaction, harmony, and tolerance. Distribution can be considered normal if the values of the skewness index are in the range from −3 to 3, and the values of the kurtosis index are in the range from −10 to 10 [39]. Since all variables met Kline’s conditions for normality of distribution, parametric statistical methods were used. Significance was set at *p* < 0.05 for all analyses.

Descriptive statistics were conducted to determine the mean values of the observed variables—subjective well-being (life satisfaction, positive and negative experiences, and flourishing), loneliness, resilience, and the quality of family functioning (cohesion, harmony, tolerance, and conflict). Pearson’s r correlation analyses were used to examine the relationship between subjective well-being (criterion) and loneliness, resilience, and the quality of family functioning (potential predictors). In order to examine the contribution of loneliness, resilience, and the quality of family functioning in explaining the aspects of subjective well-being (life satisfaction, positive and negative experiences, and flourishing), three separate multiple regression analyses were carried out.

## 3. Results

### 3.1. Participants

The study involved 86 women diagnosed with an eating disorder by a psychiatrist. The average age of the participants was 30.2 years (SD = 9.10). Of the total number of participants, 45.3% (N = 39) were diagnosed with an eating disorder during the COVID-19 pandemic (2020–2022), and 54.7% (N = 47) were diagnosed before the start of the pandemic (2000–2020). Bulimia nervosa (27.9%) was the most common diagnosis in the study, followed by binge eating disorder (25.6%), anorexia nervosa (24.4%), and unspecified eating disorder or eating disorder (22.1%).

### 3.2. Descriptive Data

In the following text, all data necessary for understanding and interpreting the aim of the study are presented. First, descriptive indicators of the variables used are presented in (Table 1). On average, participants had higher scores on the Life Satisfaction subscale, but they experience negative experiences more often and have lower levels of flourishing. The higher average score on the Loneliness Scale reflects the more frequent occurrence of such a condition among the participants. The average results on the Resilience Scale indicate that the participants are not completely sensitive but also not extremely resistant to certain life challenges. As for the quality of family functioning, the average results for all factors indicate that there is a certain degree of cohesion, harmony, tolerance, and conflict in their families, but they are not overly pronounced.

### 3.3. Main Results

A matrix of correlation coefficients of all variables included in the study is presented (Table 2). Since the three components of subjective well-being represent the three criteria of this study, the results of multiple regression analysis for each criterion are presented separately (Table 3, Table 4 and Table 5). These analyses were conducted to investigate how much of the variance in the criteria could be explained by individual psychosocial factors. Finally, the results of a qualitative analysis of data on perceived quality of support are presented.

The three aspects of subjective well-being, as expected, were interrelated, and the correlation coefficients among them were significant (medium high to high). The negative correlation between life satisfaction and positive and negative experiences implies that greater life satisfaction comes with more frequent positive experiences compared to negative ones. More frequent experience of such experiences was also associated with higher levels of flourishing. Life satisfaction was significantly correlated with loneliness, resilience, cohesion, tolerance, and conflict. The negative correlation between life satisfaction and loneliness (medium-high correlation) and conflict (low correlation) indicates that the higher the life satisfaction, the lower the feeling of loneliness and the less frequent the conflict within the family among the participants. Furthermore, greater life satisfaction in participants suggests that they are more resilient to life’s adversities and that there is a greater sense of cohesion and tolerance in their families. The variable of positive and negative experiences was significantly correlated with the variables of loneliness, resilience, cohesion, and tolerance, and it had a medium-high correlation with the variables of loneliness and resilience, while the correlation with the other two variables was somewhat lower. The correlations suggest that, with a greater number of positive experiences, the level of resilience to life’s adversities increases, as well as tolerance and cohesion in family relationships, but the level of loneliness in the research participants decreased. The variable of flourishing was significantly correlated with the variables of loneliness, resilience, cohesion, and tolerance, where higher levels of flourishing indicated a lower degree of loneliness but also stronger resilience and a higher degree of cohesion and tolerance in family relationships. When looking into the relationship among predictor variables, loneliness was significantly correlated with the variables of resilience, cohesion, and tolerance. Of the four factors on the Family Functioning scale, cohesion, tolerance, and conflict were significantly correlated, and the correlation for factors cohesion and tolerance was extremely high. The conflict factor was only significantly correlated with the harmony factor. Given the very high correlation between predictor variables cohesion and tolerance, a possible suppressor effect of one of the variables during regression analysis was checked but not proven.

The coefficient of multiple correlation between the criterion life satisfaction and the predictors loneliness, resilience, cohesion, harmony, tolerance, and conflict indicated a high correlation between the mentioned predictors and the criterion (Table 3). The obtained coefficient of determination was significant and indicated the possibility of explaining 41.9% of the variance of the criteria with the specified set of predictors. The adjusted coefficient of determination indicated that this model could predict 37.5% of the variance of life satisfaction at the population level. The predictors loneliness and resilience were the only ones with statistically significant predictive validity (*p* < 0.05), which suggests that a lower feeling of loneliness and greater resilience to life’s difficulties lead to greater life satisfaction in people with an eating disorder. In explaining the variance of the life satisfaction criteria, the other predictors did not prove to be significant.

The coefficient of multiple correlation between the criteria of positive and negative experiences and the predictors loneliness, resilience, cohesion, harmony, tolerance, and conflict indicated a high correlation between the mentioned predictors and the criterion (Table 4). The coefficient of determination was significant and indicated the possibility of explaining 44% of the variance of positive and negative experiences with the specified set of predictors. The adjusted coefficient of determination indicated that this model could predict 39.7% of the variance of positive and negative experiences at the population level. The presented results show that the predictors loneliness (*p* < 0.001), resilience (*p* < 0.01), and cohesion (*p* < 0.05) had statistically significant predictive validity, which implies that participants with more frequent feelings of loneliness, less ability to resist life’s difficulties, and with a lower sense of cohesion experience negative emotions more often than positive ones. Other predictors in the analysis did not prove to be significant in explaining the variance of this criterion.

The results of multiple regression analysis for the last criterion indicated a high coefficient of correlation between the criterion of flourishing and the set of predictors. Accordingly, from the obtained coefficient of determination, it was evident that this set of predictors could explain 54.4% of the variance for the criterion of flourishing. The adjusted coefficient of determination indicated that this model could predict as much as 51.0% of the variance in flourishing at the population level. Predictors loneliness (*p* < 0.001), cohesion (*p* < 0.01), and resilience (*p* < 0.01) significantly predicted the outcome of the criterion variable flourishing, i.e., the obtained results imply that less frequent feelings of loneliness, greater resilience to life’s adversities, and stronger feelings of cohesion in the family predict higher levels of flourishing in participants. Predictors harmony, tolerance, and conflict did not show statistically significant validity in explaining the outcome on the criterion variable.

From the presented results, it was evident that predictors loneliness and resilience had statistically significant validity for predicting outcomes for all three criteria of subjective well-being. In addition to them, in explaining variance for criteria positive and negative experiences and flourishing, the predictor family cohesion also proved to be statistically significant (Table 3, Table 4 and Table 5). Predictors tolerance and conflict, despite statistically significant correlations with criteria, did not prove to be significant in multiple regression analysis. Predictor harmony also did not prove to be statistically significant in explaining the variance of the three criteria.

**Figure 1:**
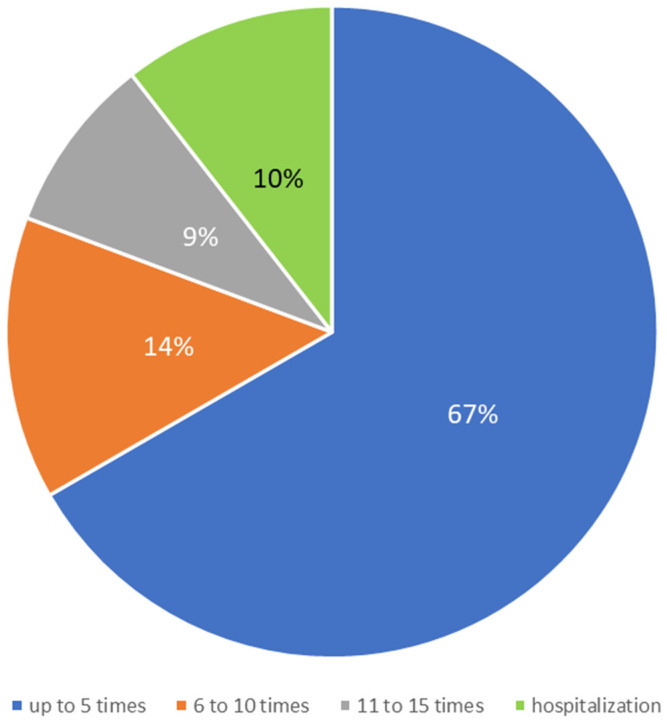
Frequency of seeking professional help among participants (N = 57).

Finally, responses from participants who once sought help from professionals related to their diagnosis of an eating disorder were analyzed. A constructed questionnaire on perceived quality of support collected data on how familiar participants were with existence of centers/associations for individuals with eating disorders near them. Of the respondents, 48.8% answered affirmatively to the posed question, 33.7% stated that there were no such institutions near them, and 17.4% were not sure if such institutions existed or not near them. Then, they were asked whether they had ever sought help from such centers or associations, and 66.3% stated that they had sought professional help. Data on how many times participants who answered positively to the previous question sought professional help were grouped into four categories (Figure 1). Participants who could not specify data on exact number reported short-term or long-term hospitalizations related to the diagnosis of an eating disorder in the last two years.

Of the 57 participants who sought help, 64.9% were satisfied with the help they received from professionals, 15.8% of participants were not satisfied with the received help, and 19.3% of participants could not assess their satisfaction with the received help.

Some participants shared their experiences and stated what they found helpful and what they would change when seeking help from professionals. What participants most often highlighted as helpful were individual and group psychotherapy, a feeling of understanding and acceptance from others, and advice from professionals; “I am being treated for disorder through individual psychotherapy privately, I would not change anything, and what helped me the most was the consistent statement of my therapist that every emotion, appearance and body shape, thought… is completely human and okay”, “I am still in the process of recovery. The new insights I have gained into my disorder and explanation of reasons for its occurrence, as well as the nutritionist group, are helpful to me”.

On the other hand, some participants reported financial constraints and lack of an adequate form of help in their environment. Furthermore, participants stated that there were long waiting lists for certain treatments, as well as large wait times between assigned appointments; “I would definitely change the frequency of appointments because they were too far apart, during physical examinations I would rather not have to listen to additional comments on my appearance and weight, additional education is needed on ‘extreme hunger’ that can occur in recovery from restrictive eating disorder”, “Nothing helped me since the first support group I was referred to was intended for much younger people than me and another program has a waiting list of about 10 months. I would change availability of such programs (more support groups) and add some day hospital/ward program intended for long-term help with eating disorders (in contrast to daily hospital wards where patients are forcibly fed without any psychological help and then sent home as soon as they gain weight”.

## 4. Discussion

This research attempts to explain the relationship between subjective well-being and various psychosocial factors in individuals with eating disorders. The study was conducted with the aim of creating a better understanding of the findings of previous research, as well as deepening this knowledge relating to the population of women diagnosed with eating disorders in the Republic of Croatia. The obtained insights are useful for understanding the background of the problems faced by individuals with eating disorders and provide insight into what is key for these individuals in coping with the diagnosis and therapeutic procedure.

### 4.1. Presence of Subjective Well-Being and Psychosocial Factors

The level of life satisfaction in participants with eating disorders in this study was slightly lower than the average theoretical level, suggesting that participants report lower levels of life satisfaction [7]. These results are consistent with findings from previous studies that reported extremely low levels of life satisfaction in women with eating disorders as well as those who have been diagnosed with an eating disorder in the past or those with specific symptoms of eating disorders [9,12]. Negative affect has been identified as the greatest precursor to eating disorders [40]. For example, experiencing abuse in childhood and exposure to traumatic events have been identified as possible risk factors for the development and maintenance of eating disorders [13]. As expected, participants achieved a slightly higher score than average on the Positive and Negative Experiences Scale. The obtained results suggest that participants more frequently experienced negative (e.g., fear, anger, sadness) compared to positive experiences in the last four weeks. This is partially consistent with previous research that reported less frequent positive experiences in individuals with eating disorders, but the frequency of negative experiences was not statistically significant [12]. More frequent negative experiences can be attributed to anxious or depressive symptoms and dissatisfaction with one’s own weight, which are often present in individuals with eating disorders [6]. Finally, as expected, the result on the Flourishing Scale was lower than the average theoretical level [7]. Such a result implies that participants express lower levels of flourishing. Lower levels were expected because flourishing encompasses an individual’s psychological needs such as competence, connectedness, and self-acceptance, the levels of which are low in individuals with eating disorders [32,41]. Such insights were confirmed by other research, where findings showed that individuals with eating disorders who have difficulty accepting and being satisfied with their body image report lower levels of flourishing [11]. Accordingly, previous research reported that girls facing the pathology of eating disorders have more difficulty with social relationships, including more conflicts and feelings of alienation from friends [42]. Since flourishing encompasses supportive and rewarding relationships, it is possible that participants in this study rated their level of flourishing lower due to a lack of such relationships [7].

Lower levels of life satisfaction and flourishing and more frequent negative experiences can be attributed to the tendency of individuals with eating disorders to perceive circumstances more negatively due to the nature of their condition and the consequences of the pandemic period. The result of participants on the Loneliness Scale did not differ from the average result, which implies that the feeling of loneliness is neither overly frequent nor rare in participants with eating disorders. More frequent feelings of loneliness were expected due to numerous findings showing higher levels of loneliness in individuals with chronic physical conditions and in individuals with numerous psychological problems [15,43]. Accordingly, it has been confirmed that the feeling of high levels of loneliness extends across the entire spectrum of severity from anorexia to overeating and obesity [17]. Also, lonelier individuals are more likely to use eating as a coping mechanism for unpleasant emotions, resulting in irregular eating habits, most commonly manifested in individuals diagnosed with binge eating disorder [18]. The results on the Loneliness Scale obtained by this study can be attributed to numerous personal and social factors that contribute to reducing or increasing feelings of loneliness, where these reasons are very subjective and differ for each individual [44]. The result on the Resilience Scale, although close to average, was slightly lower than the theoretical average, implying that participants with eating disorders still show a tendency towards lower levels of psychological resilience due to life’s adversities [45]. Lower levels have previously been recorded in individuals with eating disorders, specifically in individuals with anorexia, bulimia, and binge eating disorder [20,21]. The COVID-19 pandemic may have also affected reduced psychological resilience in participants as it has been greatly reflected on life habits and required major changes and adaptations in daily life that presented major challenges for many. Such changes strongly affected individuals with bulimia, in whom a significant worsening of symptoms has been recorded [25]. It has previously been shown that individuals with eating disorders tend to perceive their family relationships as less connected and less adaptable to their situation [30]. The results obtained were expected due to the nature of the condition of individuals with eating disorders, and it is known that the quality of family relationships, including social support and conflicts, can affect an individual’s physical as well as psychological state [46]. It is clear that family relationships play a major role in the onset and development of eating disorders, but also the nature and demands of the disorder itself can result in impaired family relationships [47,48].

### 4.2. Relationship between Subjective Well-Being and Psychosocial Factors

This study indicates mutual interrelationships between components of subjective well-being. The variables of life satisfaction and flourishing are negatively correlated with the variable of negative experiences. Such a relationship implies that more frequent negative experiences reduced feelings of flourishing and life satisfaction in participants in this study. Life satisfaction and a sense of flourishing were characterized by high positive correlation. Such results were expected because individuals who are more satisfied with life more frequently experience positive experiences and assess higher levels of flourishing in their lives [7]. Furthermore, as expected, the variable of life satisfaction was positively correlated with resilience, cohesion, and tolerance and negatively with loneliness and conflict. The negative correlation suggests that, with lower levels of loneliness and fewer family conflicts, life satisfaction increases in participants with eating disorders. The obtained significant correlations are consistent with previous research findings on the relationship between life satisfaction and resilience, family cohesion, loneliness, and conflict [27,28,32,49]. No significant correlation was found between life satisfaction and harmony. Furthermore, a significant correlation was found between the combined variable of positive and negative experiences and all factors except harmony and conflict. The obtained correlation with loneliness was in a positive direction, while the correlation with resilience, cohesion, and tolerance was in a negative direction. These findings suggest that more frequent negative experiences are associated with higher levels of loneliness and lower levels of resilience, cohesion, and tolerance. These results are consistent with previous findings that reported lower levels of resilience in individuals diagnosed with eating disorders due to exposure to life stressors and adversities [25]. The feeling of flourishing was, as expected, significantly positively correlated with resilience, cohesion, and tolerance, while it was significantly negatively correlated with the feeling of loneliness. Contrary to expectations, flourishing was not significantly correlated with harmony and conflict. The obtained significant results are consistent with previous findings, showing that, as the level of flourishing increases, there are fewer occurrences of feelings of alienation in individuals with eating disorders [42]. Also, higher levels of flourishing were recorded in individuals who assessed social and family belonging as stronger [32]. Loneliness was significantly negatively correlated with resilience and tolerance, while it had no significant correlation with harmony and conflict. This confirms earlier findings indicating higher levels of resilience in individuals with established close relationships and emotional regulation abilities [50]. Finally, a significant correlation was found among all components of the Self-Report Family Inventory except between cohesion and harmony and tolerance and harmony.

Loneliness, resilience, cohesion, harmony, tolerance, and conflict explained just under 50% of the variance of life satisfaction. Of the predictors included in the analysis, only the predictors of loneliness and resilience were significant in predicting life satisfaction, while other predictors did not show significant predictive validity for this component of subjective well-being. The significant results are consistent with previous findings that lonelier individuals report lower levels of life satisfaction [33]. In individuals with eating disorders, the feeling of loneliness is present across the entire spectrum of severity, as well as lower levels of life satisfaction, which can explain the obtained correlation between these two constructs in this study [9,17,29]. Furthermore, the significant predictive validity of resilience was also expected because resilience is one of the protective factors in individuals with eating disorders, and individuals who are more resilient to life’s adversities report greater life satisfaction [23,32]. Such results were also expected because loneliness increased and resilience declined during the COVID-19 pandemic [16,25]. Due to the role of family in the onset and course of eating disorders, it was expected that components of the quality of family functioning would significantly predict life satisfaction in individuals with eating disorders [28], especially cohesion, which has been shown to be significant in perceiving one’s own life satisfaction in individuals diagnosed with bulimia, and conflict, which has been shown to be one of the family factors that promotes symptoms of eating disorders [27,29]. Despite insufficient research on the role of family harmony and tolerance in explaining life satisfaction, their significant predictive validity was expected due to their importance in explaining the quality of family relationships.

The obtained results for positive and negative experiences met initial expectations. Based on the set of predictors in the analysis, it was possible to explain just under 50% of the variance of the variable of experienced positive and negative experiences in individuals with eating disorders. Significant predictors in experiencing positive and negative experiences were loneliness, resilience, and family cohesion. The results indicate that more frequent feelings of loneliness predict more frequent negative experiences such as anger, sadness, and fear. Such findings are not surprising considering loneliness is characterized by a lack of necessary social relationships, which is itself a very unpleasant experience for an individual [44]. Accordingly, it is known that loneliness can be the cause of numerous psychological problems such as depression and anxiety, which often occur in individuals diagnosed with an eating disorder, and the presence of such a condition reduces the possibility of more frequent positive experiences [6,12,15]. Furthermore, resilience indicates more frequent negative experiences in individuals with eating disorders, who show lower levels of resilience. Lower levels of resilience in individuals with eating disorders are not unexpected, especially during a pandemic when individuals with bulimia show a significant worsening of symptoms due to low levels of resilience [20,22,25]. The predictor of perceived cohesion in the family also proved to be significant, as expected. This relationship suggests that, with a lower perceived sense of cohesion in the family, negative experiences are more frequently experienced in individuals with eating disorders. Such a finding is consistent with previous findings showing that perceived family cohesion in childhood results in more frequent positive emotions in adulthood [34].

Furthermore, the analysis showed that, based on the set of mentioned predictors, it was possible to explain just over 50% of the variance of the flourishing. Significant predictors were loneliness, resilience, and perceived family cohesion. Lower levels of loneliness indicated a greater sense of flourishing in participants in this study. Such findings are consistent with previous research showing that lonelier individuals report lower levels of flourishing [51,52]. Flourishing encompasses psychological needs such as competence, acceptance, and self-acceptance [7]. Accordingly, individuals who lack necessary supportive relationships report lower levels of life satisfaction and flourishing [32]. The significance of the resilience predictor indicates that those with higher levels of resilience achieve a greater sense of flourishing. Such a relationship was evident in numerous resilience studies that reported that more resilient individuals tend towards optimism, recognize personal strengths and qualities, develop close relationships with others, and have developed social skills and the ability to adequately regulate emotions [50]. A predictive role of resilience in explaining flourishing was also found [32]. Perceived family cohesion was proven to be a significant predictor of the feeling of flourishing. It was previously confirmed that the quality of family relationships, including cohesion, affects an individual’s well-being in all aspects [46]. Family support and a sense of belonging affect a greater sense of subjective well-being in individuals with eating disorders [32]. In prediction of the feeling of flourishing, harmony, tolerance, and conflict were not found to be significant predictors.

Considering the perceived quality of support among participants with eating disorders, almost half of the participants reported being informed about the existence of a center/association for eating disorders near them. These data were expected considering the sample of participants for this study was collected through existing centers/associations. More than half of them, as expected, stated that they had sought professional help offered by such institutions. The largest percentage encompassed those who sought help up to five times in the last two years, and more than half of those who sought professional help reported on the usefulness of such support. The participants’ positive experiences are consistent with research showing the usefulness of various psychotherapeutic approaches in treating eating disorders, support from others, and help from professionals [28,53]. Their dissatisfaction was related to long waiting lists and the unavailability of adequate services near them, which was expected given the limited capacity of the qualified experts and centers in Croatia. Considering the findings of previous research, the insights gained from this study and the insight into the experiences of individuals with eating disorders, new paths are opened for further research on this topic and the recognition of the need for the development of new specialized institutions for individuals with eating disorders in the entire area of Croatia.

### 4.3. Limitations, Implications, and Suggestions for Future Research

Despite a better understanding of the psychosocial factors of subjective well-being in individuals with eating disorders in the Croatian sample, it is necessary to highlight the methodological limitations of this study. The first methodological shortcoming is related to the sampling method of this cross-sectional online study. Namely, the collected sample of research participants was convenient. Although one should not diminish the consequences that the pandemic has had on individuals with eating disorders, this study and the instruments used do not provide a clear insight into whether the levels of the three components of subjective well-being and other factors are based on events related to the pandemic or current life situations that individuals with eating disorders are going through. Also, for a better understanding of eating disorder issues, it is recommended to conduct statistical analyses for each eating disorder in order to collect data on differences in subjective well-being among individuals with different diagnoses of eating disorders. Finally, as far as the external validity of the research is concerned, one should consider the fact that most participants completed the research during the holiday period, which is characterized by the easy availability of numerous food products and the highlighting of their nutritional values, which could have affected their perception of subjective well-being and feelings of loneliness. Since this study is correlational, it excluded the possibility of controlling research conditions, as well as the existence of a control group, and therefore cannot provide conclusions about causal relationships between variables, which reduces the possibility of valid statistical conclusion. Future research should address the aforementioned limitations but also explore additional cultural and psychosocial factors that could possibly have an impact on the onset and progress of eating disorders.

Despite these limitations, by reviewing previous research and obtaining findings from this study, useful data have been obtained that can have certain theoretical and practical implications. This work has provided data on significant predictors of subjective well-being in individuals with eating disorders that confirm previous findings but also create opportunities for improving and expanding these insights. Given that treatment of eating disorders is very challenging and long lasting, these insights to some extent facilitate recognition of life aspects that may precede the development of the disorder, based on which professionals can determine what to focus on in treatment process. The results can be used to educate professionals in all areas of health care, but also the general public, about situations that may precede and affect eating disorders, as well as possible protective factors for such condition. By educating the general public about the seriousness of eating disorders, the stigma associated with such conditions is reduced, which can encourage and motivate individuals with eating disorders to seek professional help. Educating professionals is extremely important given the necessity for a multidisciplinary team in treating the symptoms of eating disorders. Such findings can be used to design preventive programs that work on strengthening resilience and self-image and strengthening close relationships with the goal of increasing subjective well-being, which can prevent further development of symptoms of eating disorders. By understanding the dynamics between feelings of loneliness and the development of eating disorder symptoms, paths are opened for the development of numerous preventive programs aimed at recognizing the importance of creating social support and empowering it. Given the complexity of the problem of loneliness and feelings of social isolation, it would be best to implement such programs in schools, which play a major role in nurturing and developing formal and informal relationships. The goal of encouraging peer support and assistance is to achieve understanding and acceptance from peers but also to achieve higher levels of subjective well-being, creating a more positive self-image and self-acceptance in individuals. The insights gained can also be useful to families of individuals with eating disorders, helping them to recognize their needs and their own role in the treatment process but also in the onset of eating disorder symptoms. In addition, it encourages the implementation of the insights gained in the development of programs for various centers and associations for individuals with eating disorders.

## 5. Conclusions

The aim of this study was to determine the perceived quality of professional support, the relationship between subjective well-being (life satisfaction, positive and negative experiences, and flourishing), loneliness, resilience, and family functioning quality, and the possibility of predicting subjective well-being based on knowledge of psychosocial factors in individuals with eating and feeding disorders. This study found lower levels of life satisfaction in participants with eating disorders, who reported more frequent negative experiences and lower levels of flourishing. Participants showed slightly lower levels of psychological resilience and reported lower levels of cohesion, harmony, tolerance, and conflict in family relationships. The obtained findings suggest that more frequent negative experiences are associated with higher levels of loneliness and lower levels of resilience, cohesion, and tolerance. The feeling of flourishing is significantly positively correlated with resilience, cohesion, and tolerance, while it is significantly negatively correlated with the feeling of loneliness.

The best predictors of life satisfaction in individuals with eating disorders are loneliness and resilience; experienced negative and positive experiences are best predicted based on loneliness, resilience, and perceived family cohesion.

The study also provided insight into the number of participants who sought professional help and shared their experiences about what was helpful from professionals and what was not. Our findings can help experts with the detection of the psychosocial factors that can be present in eating disorders.

## Figures and Tables

**Table 1:** Descriptive indicators of subjective well-being, loneliness, resilience, and the quality of family functioning (cohesion, harmony, tolerance, and conflict) (N = 86).

	N	M	SD	Min	Max	KS
Life satisfaction	86	19.24	6.69	6.00	32.00	0.102 *
Positive and negative experiences	86	2.60	9.24	−17.00	21.00	0.064
Flourishing	86	35.1	10.93	9.00	56.00	0.082
Loneliness	86	49.80	10.02	26.00	68.00	0.083
Resilience	86	2.60	0.84	1.00	4.67	0.074
Cohesion	86	2.90	0.96	1.20	4.70	0.070
Harmony	86	2.90	0.42	1.86	4.00	0.105 *
Tolerance	86	3.19	1.21	1.00	5.00	0.096 *
Conflict	86	2.83	0.78	1.25	4.25	0.067

Legend: * *p* < 0.05; M—mean; SD—standard deviation.

**Table 2:** Pearson correlation coefficients of the variables of loneliness, resilience, cohesion, harmony, tolerance, and conflict with aspects of subjective well-being (N = 86).

	1.	2.	3.	4.	5.	6.	7.	8.	9.
1. Life satisfaction		−0.596 **	0.728 **	−0.454 **	0.383 **	0.509 **	0.165	0.488 **	−0.266 *
2. Positive and negative experiences			−0.652 **	0.516 **	−0.459 **	−0.408 **	−0.079	−0.313 **	0.163
3. Flourishing				−0.614 **	0.436 **	0.482 **	0.127	0.402 **	−0.149
4. Loneliness					−0.336 **	−0.312 **	−0.016	−0.298 **	0.153
5. Resilience						0.234 *	−0.099	0.224 *	−0.193
6. Cohesion							0.073	0.910 **	−0.557 **
7. Harmony								0.066	0.332 **
8. Tolerance									−0.570 **
9. Conflict									

Legend: * *p* < 0.05, ** *p* < 0.01.

**Table 3:** Results of multiple regression analysis for the criterion life satisfaction (N = 86).

	Unstandardized Coefficients		Standardized Coefficients			
Model	B	Std. Error	β	t	*p*	
Constant	9.680	6.790		1.426	0.158	
Loneliness	−0.176	0.063	−0.263	−2.788	0.007	
Resilience	1.785	0.743	0.223	2.404	0.019	
Cohesion	1.755	1.462	0.253	1.200	0.234	
Harmony	2.811	1.559	0.175	1.803	0.075	
Tolerance	0.492	1.170	0.089	0.420	0.675	
Conflict	−0.427	1.016	−0.050	−0.420	0.675	
R	R^2^	Adjusted R^2^	S_e_		F	*p*
0.648	0.419	0.375	5.286		9.507	<0.001

**Table 4:** Results of multiple regression analysis for the criterion of positive and negative experiences (N = 86).

	Unstandardized Coefficients		Standardized Coefficients			
Model	B	Std. Error	β	t	*p*	
Constant	8.398	9.210		0.912	0.364	
Loneliness	0.321	0.085	0.348	3.756	0.000	
Resilience	−3.355	1.007	−0.303	−3.331	0.001	
Cohesion	−5.599	1.984	−0.585	−2.823	0.006	
Harmony	−1.616	2.114	−0.073	−0.764	0.447	
Tolerance	2.855	1.587	0.375	1.800	0.076	
Conflict	−0.434	1.378	−0.037	−0.315	0.754	
R	R^2^	Adjusted R^2^	S_e_		F	*p*
0.663	0.440	0.397	7.170		10.337	<0.001

**Table 5:** Results of multiple regression analysis for the criterion of flourishing (N = 86).

	Unstandardized Coefficients		Standardized Coefficients			
Model	B	Std. Error	β	t	*p*	
Constant	28.939	9.829		2.944	0.004	
Loneliness	−0.486	0.091	−0.445	−5.332	0.000	
Resilience	3.111	1.075	0.238	2.894	0.005	
Cohesion	6.234	2.117	0.550	2.945	0.004	
Harmony	2.054	2.256	0.078	0.910	0.365	
Tolerance	−2.019	1.693	−0.224	−1.192	0.237	
Conflict	1.644	1.470	0.117	1.118	0.267	
R	R^2^	Adjusted R^2^	S_e_		F	*p*
0.738	0.544	0.510	7.652		15.728	<0.001

## Data Availability

The data presented in this study are available on request from the corresponding author.
